# **Lip Reconstruction after Tumor Ablation**

**Published:** 2016-01

**Authors:** Ali Ebrahimi, Mohammad Hossein Kalantar Motamedi, Azin Ebrahimi, Mohammad Kazemi, Amin Shams, Haleh Hashemzadeh

**Affiliations:** 1 Department of Plastic Surgery, Trauma Research Center, Baqiyatallah University of Medical Sciences, Tehran, Iran; 2 Department of Oral and Maxillofacial Surgery, Trauma Research Center, Baqiyatallah University of Medical Sciences, Tehran, Iran; 3 Medical student,Tehran University of Medical Sciences, Tehran, Iran; 4 Trauma Research Center, Baqiyatallah University of Medical Sciences, Tehran, Iran; 5 Private Practitioner, Tehran, Iran; 6 Dental Research Center, Tehran University of Medical Sciences, Tehran, Iran

**Keywords:** Lip, Reconstruction, Tumor, Ablation

## Abstract

Approximately 25% of all oral cavity carcinomas involve the lips, and the primary management of these lesions is complete surgical resection. Loss of tissue in the lips after resection is treated with a variety of techniques, depending on the extension and location of the defect.

Here we review highly accepted techniques of lip reconstruction and some of new trials with significant clinical results. Reconstruction choice is primarily depend to size of the defect, localization of defect, elasticity of tissues. But patient’s age, comorbidities, and motivation are also important. According to the defect location and size, different reconstruction methods can be used. For defects involved less than 30% of lips, primary closures are sufficient. In defects with 35–70% lip involvement, the Karapandzic, Abbe, Estlander, McGregor or Gillies’ fan flaps or their modifications can be used. When lip remaining tissues are insufficient, cheek tissue can be used in Webster and Bernard advancement flaps and their various modifications. Deltopectoral or radial forearm free flaps can be options for large defects of the lip extending to the Jaws. To achieve best functional and esthetic results, surgeons should be able to choose most appropriate reconstruction method. Considering defects’ size and location, patients’ expects and surgeon’s ability and knowledge, a variety of flaps are presented in order to reconstruct defects resulted from tumor ablation. It’s necessary for surgeons to trace the recent innovations in lip reconstruction to offer best choices to patients.

## INTRODUCTION

The upper and lower lips are distinct facial anatomic parts form majority features of lower 1/3 of face, and their functional and aesthetic importance is undeniable. Although over 200 methods have been described for reconstruction of lip defects since 1000 BC and many have been considered to be ideal, none of them is perfect or suitable for the reconstruction of all kinds of defects.^1^^-^^4^

Defects of lips may occur as a result of hereditary disorders (such as cleft lip), trauma and tumors. Defects of the lips are more frequently encountered because of the high incidence of tumors of lips. Squamous Cell Carcinoma (SCC) is the most common malignancy related to the lips (95%) and the lower lip more commonly involved in comparison with the upper lip (9:1). Loss of tissue in the lips is treated with a variety of techniques, depending on the extension and location of the defect.^5^ Tumors of the lips should be treated with surgery and postoperative radiation when there are poor prognostic indicators that include multiple levels of positive lymph nodes, extra capsular extension of the cancer in lymph nodes, deep invasion of the primary tumor, neural and vascular invasion, tumor margins less than 5 mm.^6^

*Goals of Reconstruction*

The aims of reconstruction should be to maintain oral competence, maintain maximum oral aperture, maintain mobility, maintain sensation when possible, and maximize aesthetic result. For example a reduction to less than 50% of stoma size produces oral dysfunction, especially for those who wear dentures.^7^^,^^8^ For lip reconstruction, first we should assess the lesion to determine the amount of mucosa, muscle, and skin that will be involved before incisions are made.

Classifications of lip defects are based on the anatomic location (skin, vermillion, or both), thickness (partial thickness or full thickness), and width of the defect relative to the overall width of the lip.^9^ Using this defect based approach, defects can be divided into vermilion only defects, partial-thickness (cutaneous) defects, defects less than one third of the total lip length, defects between one third and two thirds total lip length, and total lip defects.^10^^,^^11^

In order to reconstruct the lips following tumor ablation, the “reconstruction ladder” starts with the simplest procedures, moving up to the most complex. Primary closure of the lip is the simplest technique for small defects. The next steps of reconstruction are local flaps, and these have the advantage of good color match, easy accessibility, simplicity in the surgical technique, and use of innervated muscle for function. Local flaps also have some disadvantages, including the need to make extra skin incisions on the face and the lack of sufficient tissue for major defects.^11^^,^^12^

Free flaps and grafts are the next choices for reconstruct ion of post tumor excision lip defects.

As a result of the relatively lower incidence of cancer, the reconstructive techniques for defects of the upper lip are fewer than lower lip. Based on classification was mentioned before, we will review current trends for lip reconstruction:

*Vermilion Defects*

The vermillion is the most prominent feature of the lip. The preferred method for repair of vermillion-only defects is an advancement of labial mucosa, which is then re-draped over the underlying orbicularis musculature.^2^^,^^9^ The plane of dissection is between the uninvolved mucosa and underlying musculature.^13^ When simple advancement is not sufficient, other techniques can be used. These include mucosal V-Y advancement flaps, cross-lip mucosal flaps, sliding vermilion advancement flaps, and tongue flaps. The cross-lip mucosal flaps and tongue flaps require staged secondary surgery after two or three weeks.^14^

When vermilion replacement requires more bulk or the oral mucosa is not available, anterior-based tongue flaps are useful and when using W-shaped excisions in young patients where disguising defects is more difficult, a design that places the W limbs in the submental fold can be considered.^11^ Sand *et al.*,^15^ in a recent study comparing vermillion mucosal advancement flaps with direct primary closure of vermillion defects, observed that mucosal advancement flaps resulted in better maintenance of vermillion width compared with primary closure. Mucosal cross-lip flaps use the labial mucosa from one lip to reconstruct a vermillion defect of the opposing lip. A novel technique has been described by Manafi et al as Mutual cross lip musculomucosal flaps for correction of major vermillion defects which is very helpful in cases of lip hemangioma.^16^ The major disadvantage of these flaps is that the reconstruction must be staged, with pedicle division occurring 2 or more weeks after initial flap positioning. 

*Cutaneous Defects*

Cutaneous defects are best reconstructed with local flaps from adjacent cutaneous lip tissue, where incisions placed in the borders of aesthetic subunits.^9^ Partial-thickness perilabial defects can be closed either by primary closure or with local transposition flaps.^2^ Primary closure is the ideal reconstructive option, with the long axis of the fusiform excision placed within the perioral relaxed skin tension lines, which tend to be perpendicular to the horizontal axis of the lip.^1^^,^^9^

When local tissue transfer is used, only outer skin and subcutaneous tissue are involved; the facial musculature will remain intact. Cutaneous lower lip can be reconstructed with a variety of flaps from the chin and submandibular area. Because the chin is a very visible aesthetic unit, incisions should be planned where there is minimal chin distortion. Larger lower lip defects reconstruction can be done using an inferiorly based or bilateral inferiorly based melolabial flaps.^13^

Upper lip cutaneous defects can be reconstructed with rotation advancement flaps or transposition flaps.^1^^,^^9^^,^^13^^,^^17^ A laterally based transposition flap is good for small cutaneous defects in the lower portion of the upper lip, as described by Converse.^18^ Larger defects which is adjacent to the vermilion can be closed using large laterally based advancement flaps.^13^

*Full-Thickness Defects Less Than One Third Total Lip Length*

The normal lip width is about 7 cm, and defects with size between 2 and 2.5 cm can be closed primarily perfectly most of times.^11^^,^^19^ Full-thickness defects require reconstitution of skin, muscle, and mucosa. When defects involve less than one-third of the lip, primary closure is usually possible without following “tight lip” or significant microstomia.^11^ Wedge-shaped or V-shaped excisions with primary closure can be performed in upper lip defects less than one third the length of the lip and up to one half the length in lower lip defects. When planning the excisions, the apex of the excision should not extend past the mental crease (for lower lip lesions) or melolabial crease (upper lip lesions).^1^

When using W-shaped excisions for larger defects, it is important not to suture across the tip of the small inverted V-shaped flap at the inferior portion of the excision to avoid avascular necrosis of the tip. In this circumstance a suture is passed through the skin of one edge of the defect, and then passed through the subdermal layer of the tip of the V-shaped flap and out from deep through the skin on the opposite edge. This suture will secure the tip of the V shaped flap in place without compressing the skin of the flap.^11^

The vermilion should preferably be marked prior to incision to prevent distortion of this important aesthetic junction that occurs during injection and subsequent surgical dissection. It is also important to remember that primary closure of these defects should not result in any appreciable microstomia. If this occurs, a secondary reconstruction option should be used. Advancement flaps are best suited for correcting central defects of the upper and lower lips. The reconstructed lip tends to be tighter than the opposing lip, which may be more pronounced when the lips are together. This effect will generally decrease over time.^1^^,^^2^

It’s worthy to mention that a V-Y advancement flap is an excellent technique for reconstructing lateral, skin-only lip defects larger than 1 cm^2^. This method places scars along aesthetic borders and advances skin of similar characteristics into the upper lip. Furthermore, this method maintains oral sphincter competence and facial nerve function.

*Full-Thickness Defects between One Third and Two Thirds Lip Length*

**Fig. 1:**
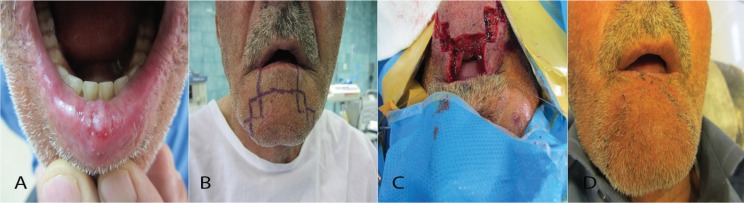
A 67-year patient was diagnosed as squamous cell carcinoma. The post-surgery defect was reconstructed via Step-Ladder flap, A. Pre-operation image, B. Flap design, C. Intra operation Incisions, D. Two weeks after reconstruction

The reconstruction of defects greater than one third of lips usually requires more complex technique than primary closure. Defects that are between one third and two thirds the total lip length represent the most complex decision making challenge for the reconstructive surgeon. The stepladder technique has shown excellent results (Figure 1). This technique can be applied unilaterally, bilaterally, or combined with vermilionectomy or other flaps.^11^ Two other major techniques are available to reconstruct these defects: transoral cross lip flaps (Abbe and Estlander) and circumoral advancement rotation flaps (Karapandzic and Gillies).^2^^,^^20^

Like Other defects, many patient-related and defect-related issues must be taken into consideration during treatment planning. These include the patient’s age, previous treatment, the presence or absence of dentition (need for dentures), the size of the soft tissue defect including individual lip subunits and adjacent structures (chin, melolabial creases), the need for a one-stage or two-stage procedure, and tissue laxity. The primary goals of reconstruction, the restoration of function and form, must always be taken into consideration as well.

Hanasono and Langstein^21^ suggest defects between one third and two thirds of the lip with sufficient lip tissue and commissure involvement can be closed with the Karapandzic as the first choice and the Estlander as secondary choice. When the commissure is not involved, the defect may be closed with the Karapandzic or the Abbe. In 2011, an extended Karapandzic flap technique was described by Sood *et al. *in patients with larger defects as an alternative to micro-vascular free flaps and regular Karapandzic flaps with good results.^7^

**Fig. 2:**
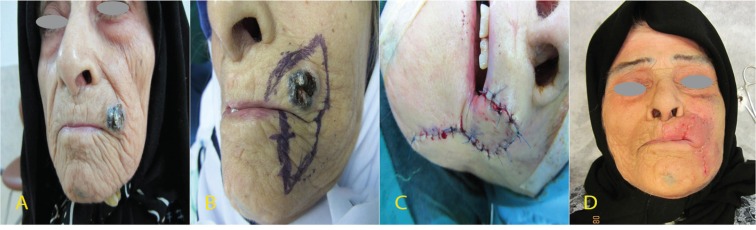
Abbe-Estlander flap in a 59-year patient diagnosed by upper lip squamous cell carcinoma, A. Pre-operation image, B. Flap design, C. Immediate post-operation image, and D. 10 days after surgery

**Fig. 3:**
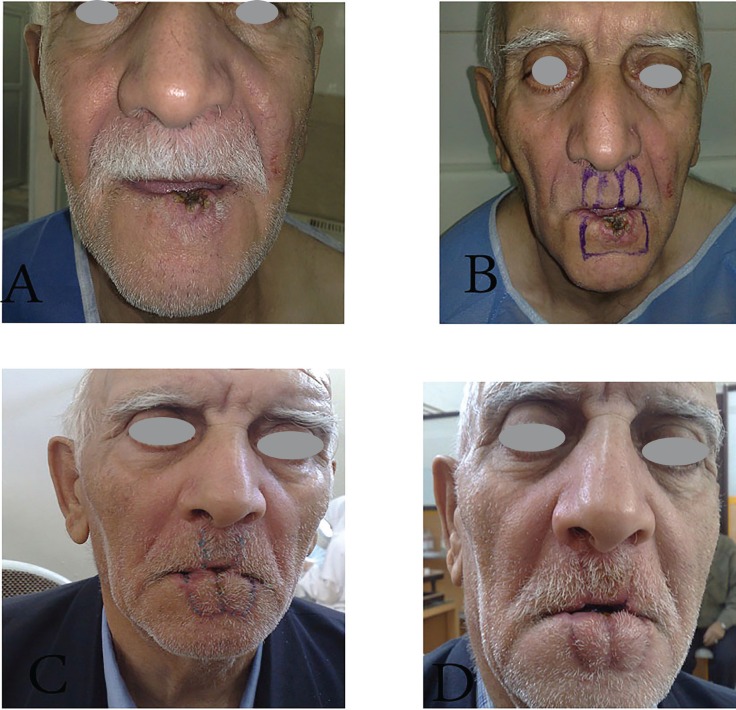
A 71 years old man with large squmous cell carcinoma of lower lip, A. Before operation, B. Designing of bilateral Abbe flap for reconstruction of lower lip, C. Early view after tumor excision and reconstruction, D. Postoperative view after three months

The Abbe flap is used for defects medial to the commissure, whereas the Estlander flap incorporates the commissure in the design (Figure 2). The Abbe flap requires division of the pedicle, which crosses the oral stoma, after 2 to 3 weeks. A better proportion between the upper and lower lips can also be maintained with this flap (Figure 3). However, the main disadvantages of this method are the fact that it is a two-stage procedure and that there is a possibility of complete loss of the flap.^22^

The reconstruction of the lips following the tumor ablation with Abbe flap or its modifications may not be always satisfactory because it leaves parallel scars extending to the free vermilion margin, a trapdoor deformity and leave a “cleft lip like” appearance. Genc *et al.* presented a process to reconstruct lower lip with an Abbe–Estlander flap modification that preserved the same side vascular pedicles and they believe it is a more advantageous method in terms of feeding of the flap according to the conventional Abbe–Estlander flap for patients who have undergone neck surgery. Furthermore, the superior labial artery is backed up for the future life of the patient for any procedure requiring its use.^23^

The Estlander flap can be additionally modified by designing the flap to lie within the melolabial crease, thus decreasing the prominence of the donor site incision.^2^ This technique involves a full-thickness (3 layers) lip-switch flap based on the labial artery. The flap is rotated 180 and sutured into the defect of the lower lip, allowing for restoration of perioral competence. Both upper and lower lip commissure reconstruction can be performed using this technique. Often a secondary revision commissuroplasty will be necessary to improve aesthetics because of resulted commissure blunting.^11^

**Fig. 4:**
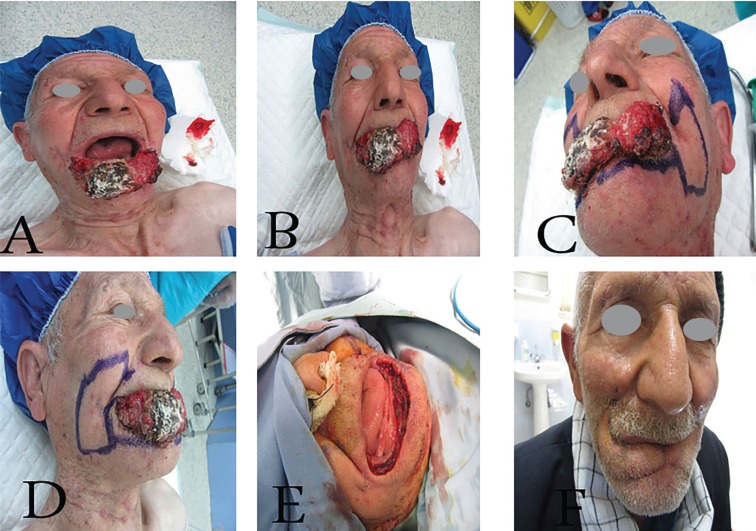
A 70 years old man with large squmous cell carcinoma of lower lip,A,B- before operation,C,D-designing of bilateral Gillie’s “fan flap”and tongue flap for reconstruction of total lower lip and vermilion E-intraoperative view after tumor excision F-postoperative view three months postoperation

One drawback of these flaps (Abbe, Estlander) is that they are denervated. Sensory functions may return after several months, usually in this order: pain, touch, and temperature. Hypersensitivity of the flap is a complication sometimes happen, but will be resolved by the first year. Gillie’s “fan flap” is a modification of the cross-lip technique that advances the ipsilateral remaining lip segment with a portion of the opposing ipsilateral lip (Figure 4).^1^

Although fan flaps work well, especially for cancers close to the commissure, they are often more difficult to design and have a tendency to become edematous, resulting in a “pincushion” deformity because of their small pedicle. The submental island flap is a useful locoregional flap that can help in the reconstruction of lip defects. It provides the advantages of being a reliable flap with an axial-pattern blood supply, with excellent color match, tissue thickness, and ease of harvest.^11^

**Fig. 5:**
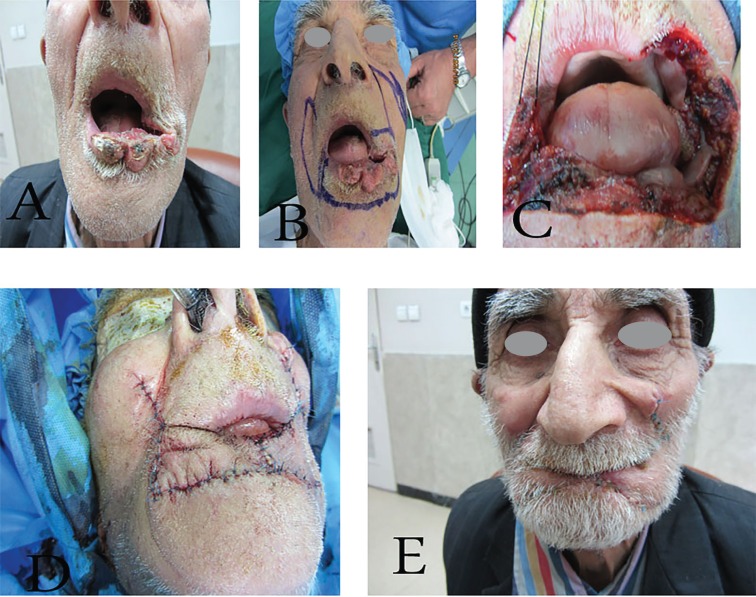
A 74 years old man with squmous cell carcinoma of lower lip and commissure involvement, A. Before operation, B. Designing of right Karapandzic flap and left cheek flap for reconstruction of total lower lip and commissure, C. Intraoperative view after tumor excision, D. Postoperative view, E. One month post-operation

The Karapandzic flap is an advancement-rotation flap that maintains lip mobility and sensation provides excellent oral competence.^2^ The original technique involved extending the incisions parallel to the upper lip toward the nasal ala, medial to the nasolabial fold. It is more commonly used for lower lip defects but can also be applied to defects of the upper lip (Figure 5). Sensation and mobility are maintained through meticulous dissection of the neurovascular structures entering the flap between the orbicularis oris musculature and surrounding facial musculature and soft tissue.

The advantages of this method are that the flap is fully innervated, so there is preservation of sensation and motor function, and it can be used to seal large defects with similar, adjacent tissue.5 Another advantage is that it is a one-stage procedure. The disadvantages are that the lip circumference is reduced and can lead to microstomia and there is rounding or distortion of the commissures. In our experience, one third of patients had a commissuroplasty to correct the microstomia that was a complication of the Karapandzic flap.^5^

*Total Lip Defects*

**Fig. 6:**
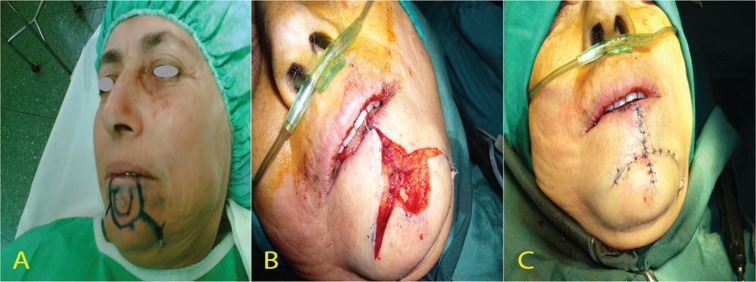
Large lower lip defect was reconstructed by a Bernard von Burrow-Webster flap, A. Flap design, B. Intra operation image, C. Post operation Image

When the defect involves more than 80 percent of lip, reconstruction will be challenging.^24^ Although several techniques have been described for total lip reconstruction, it is still difficult to reach a certain reconstructive plan, which often requires lengthy and risky microsurgical procedures.^25^ Most patients with these defects will have significant dysfunction and disability. The two major available techniques are local flap reconstruction (Bernard von Burrow – Webster technique) (Figure 6) and free microvascular tissue transfer.^2^

Defects of more than two thirds of the lower lip with sufficient adjacent tissue may be closed with the Karapandzic or the Bernard-Burow flap. When there is insufficient adjacent tissue, a free flap may be used to close the defect.^7^ The Bernard–von Burow–Webster technique is an advancement flap with the excision of cutaneous triangles. Upper lip reconstruction is accomplished with the excision of four triangles of cheek skin, and lower lip reconstruction with the excision of three triangles. Although these were originally described as full-thickness excisions, these are generally performed as cutaneous excisions only.^6^

Reconstruction with this technique is a dynamic and results in drooling. Additionally, any mucosal defect must be repaired separately because the reconstruction is cutaneous only. This technique is probably better suited for upper lip reconstruction, because there is less risk for the development of postoperative oral incompetence.^11^ Hamahata *et al.* stated Webster technique is mostly used for lower lip defects greater than 80% of the lip; with several minor modifications having been reported.^26^^-^^28^

However, resulting scars in the chin area (Schuchardt flap, a half-circle scar) are relatively conspicuous in Asian populations because of the trapdoor deformity. On the other hand, Johanson staircase flap technique, which is used to reconstruct lower lip defects of up to two thirds of the lip, results in relatively inconspicuous scarring and prevents trapdoor deformity.^29^ In 1974, Johanson *et al.*^30^ reported their staircase flap technique to reconstruct lower lip defects up to two thirds in size. But it can be used for less than 80% lower lip defects with or without an additional flap. For a near-total lower lip defect, techniques such as Gillies fan flap and the Karapandzic^21^^,^^31^ method have been described. 

It has been reported that free flap successfully was used in total lip reconstruction with excellent results.^32^^-^^35^ However, the free flap is largely limited about oral function and aesthetic results because after all, donor site tissue is different from oral tissue. Even if the free flap reconstruction has steadily been improved, the result of free flap reconstruction is still a dysfunctional dam and different texture from facial skin.^29^

When the lip is tight and patient doesn’t have lax micro-vascular flaps should be discussed and considered, and this holds especially true if the tissues are already scarred or contracted locally (eg, after radiation).^11^ Micro-vascular reconstruction allows for a single-stage reconstruction of total lip defects. This allows for reconstruction of a large tissue area, including affected areas of cheek and/or chin. For reconstruction of the entire lower lip, fasciocutaneous flaps (e.g., radial forearm and anterolateral thigh flaps) have proven to be reliable.^36^^-^^39^

The use of free revascularized osteo-cutaneous flaps (e.g., fibula or iliac crest flaps) permits reconstruction of large composite defects of the lip and mandible.^40^ Potential complications of total lip reconstruction include hypertrophic scarring, disfigurement, loss of sensation, potential microstomia, loss of oral competence, and loss of the natural gingivobuccal sulcus. This in turn can result in decreased ability to eat or at the very least cause a substantial alteration in the patient’s diet. Meticulous surgical technique and preoperative planning can help to minimize these untoward effects.

Ozdemir *et al.*^41^ reported on 17 patients who underwent reconstruction with sensate radial forearm flaps. The results were similar to those of Jeng *et al.*^37^ Also, they demonstrated return of touch sensation, temperature sensation, and two-point discrimination 4 months postoperatively in 16 of 17 patients.^41^ Reconstruction of both upper and lower lips is extremely. Upper and lower lip reconstruction via microsurgery has been reported. Jallali and Malata reported cases of total loss of the upper and lower lips reconstructed with a free vertical rectus abdominis flap for patients with fulminant pneumococcal septicemia.^42^

Daya used free radial forearm palmaris longus tendon and brachioradialis chimeric flap to reconstruct both upper and lower lips.^43^ Nthumba and Carter reported the simultaneous reconstruction of upper and lower lips by using a combination of platysma flaps and deltopectoral flaps and provided mucosal lining and a scalp visor flap.^44^ Although surgical methods for free flap reconstruction of the oral circumference have improved,^38^^,^^45^ it is still present limitations, for example, flap numbness, drooling and oral competence. Free radial forearm flap combined with Palmaris longus often are used, but such reconstruction results in adynamic and non-sensory reconstruction.^25^

Other options for reconstruction of partial or total lip defects have been reported. These include the pectoralis major myocutaneous flap, deltopectoral flap, cervicodeltopectoral flap, and temporal forehead flap.^1^^,^^46^ A nice alternative to the radial forearm flap for the reconstruction of large 3-layer lip defects is the anterolateral thigh flap. One of the primary advantages of the anterolateral thigh flap lies specifically in the donor site, because it does not require a split skin graft for coverage and can be closed primarily, unlike the radial forearm flap.^40^^,^^47^

*Adjunctive Procedures*

Sometimes a commissuroplasty is needed to correct the asymmetry which resulted from oral commissure reconstruction.^1^ Care must be taken during commissuroplasty to avoid excessive disruption of the orbicularis musculature that could result in oral incompetence.^13^

## CONCLUSION

The lips play a key role in facial esthetics. So, to achieve best functional and esthetic results, surgeons should be able to choose most appropriate reconstruction method. In the process of treatment planning, surgeons must consider the defect’s characteristics such as: the remaining tissue after tumor ablation, skin laxity and, the most important, patient’s decision. It’s necessary to involve patient in decision making process because these reconstruction procedures most of the time can be challenging and esthetic results could be far from patient’s satisfaction. 

In the subject of reconstruction, local flaps show most promising results either on aesthetics or functions. But considering defect size and location, patients’ expects and surgeon’s ability and knowledge a variety of flaps are presented in order to reconstruct defects resulted from tumor ablation. In this article, we reviewed the most common reconstruction methods performed for years and also try to introduce some new techniques that can be helpful. It’s necessary for surgeons to trace the recent innovations in lip reconstruction to offer best choices to patients.

## CONFLICT OF INTEREST

The authors declare no conflict of interest.
